# The impact of bicuspid valve morphology on the selection of transcatheter aortic valve implantation devices: an *in silico* study

**DOI:** 10.1093/ehjimp/qyaf018

**Published:** 2025-02-05

**Authors:** Benedetta Grossi, Giulia Luraghi, Sara Barati, Chiara Forte, Luca Gerosa, Ottavia Cozzi, Fabrizio D’Ascenzo, Gianluigi Condorelli, Francesco Migliavacca, Giulio Stefanini

**Affiliations:** Department of Biomedical Sciences, Humanitas University, Via Rita Levi Montalcini 4, Pieve Emanuele, Milan 20072, Italy; Department of Chemistry, Materials and Chemical Engineering, Politecnico di Milano, Piazza L. da Vinci 32, Milan 20133, Italy; Department of Chemistry, Materials and Chemical Engineering, Politecnico di Milano, Piazza L. da Vinci 32, Milan 20133, Italy; Department of Chemistry, Materials and Chemical Engineering, Politecnico di Milano, Piazza L. da Vinci 32, Milan 20133, Italy; Department of Chemistry, Materials and Chemical Engineering, Politecnico di Milano, Piazza L. da Vinci 32, Milan 20133, Italy; Department of Chemistry, Materials and Chemical Engineering, Politecnico di Milano, Piazza L. da Vinci 32, Milan 20133, Italy; Department of Biomedical Sciences, Humanitas University, Via Rita Levi Montalcini 4, Pieve Emanuele, Milan 20072, Italy; Cardio Center, IRCCS Humanitas Research Hospital, Via Alessandro Manzoni 56, Rozzano, Milan 20089, Italy; Division of Cardiology, Cardiovascular and Thoracic Department, Azienda Ospedaliero-Universitaria Città della Salute e della Scienza di Torino, Corso Bramante 88, Turin 10126, Italy; Department of Biomedical Sciences, Humanitas University, Via Rita Levi Montalcini 4, Pieve Emanuele, Milan 20072, Italy; Cardio Center, IRCCS Humanitas Research Hospital, Via Alessandro Manzoni 56, Rozzano, Milan 20089, Italy; Department of Chemistry, Materials and Chemical Engineering, Politecnico di Milano, Piazza L. da Vinci 32, Milan 20133, Italy; Department of Biomedical Sciences, Humanitas University, Via Rita Levi Montalcini 4, Pieve Emanuele, Milan 20072, Italy; Cardio Center, IRCCS Humanitas Research Hospital, Via Alessandro Manzoni 56, Rozzano, Milan 20089, Italy

**Keywords:** TAVI, finite element method, patient-specific simulations, bicuspid aortic valve

## Abstract

**Aims:**

Bicuspid aortic valve (BAV) represents a challenge for transcatheter aortic valve implantation (TAVI). Few data are reported about the procedural implications of BAV using different self-expandable devices. The aim of this study is to investigate how BAV and tricuspid aortic valve (TAV) morphologies influence device selection and their impact on the potential development of post-operative conduction disturbances, using a novel *in silico* approach.

**Methods and results:**

Five patients with BAV undergoing TAVI were enrolled. TAVs were virtually modelled within each BAV patient-specific anatomy, resulting in 10 virtual patients. Acurate Neo2 and Evolut R implantations were subsequently simulated across all cases. Post-implantation stresses exerted on both the stent and aortic root were measured, allowing a comparative analysis of the impact of the two valve morphologies. Comparing stent stresses between BAV and TAV configurations, the stress gap increased by 21.96 ± 5.35% (*P* = 0.01) in Acurate Neo2 cases (*n* = 6) compared with Evolut R cases (*n* = 4). The analysis of aortic root stresses showed no significant differences between BAV (*n* = 5) and TAV (*n* = 5) configurations, with a mean stress difference of 5.1 ± 8.17% (*P* > 0.05).

**Conclusion:**

Our patient-specific model shows that high radial force devices, such as Evolut R, demonstrate consistent expansion regardless of valve morphology, without increasing the risk of post-implantation conduction disturbances, hence resulting more suitable for BAV cases. Incorporating this methodology into pre-operative planning could support clinicians in selecting the most suitable device with a patient-specific approach.

## Graphical Abstract

**graphical abstract:**
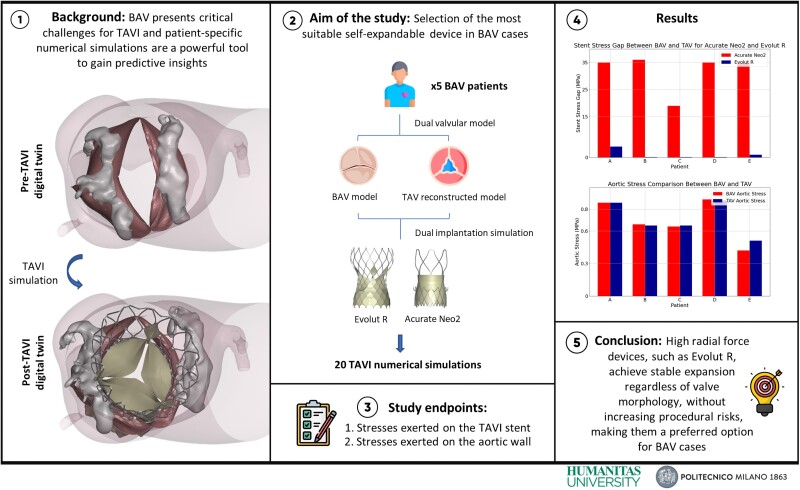

## Introduction

Bicuspid aortic valve (BAV) stands as the most prevalent congenital heart abnormality, impacting nearly 2% of the population. To categorize the diverse origins of BAV, Sievers’ classification method is commonly employed.^1^ Specifically, Sievers delineated three BAV categories: (i) Type 0, composed of two natural leaflets; (ii) Type 1, characterized by a single raphe between two adjacent cusps; and (iii) Type 2, featuring two raphes. Notably, Type 1 BAV constitutes the most prevalent subtype, accounting for approximately 90% of affected individuals.^2^

The altered biomechanics and haemodynamics associated with BAV contribute to increased mechanical strain and abnormal wall shear stress, accelerating the deposition of calcifications. Consequently, individuals with BAV face a heightened risk of developing aortic stenosis (AS). Notably, patients with BAV are prone to developing AS a decade or two earlier than those with tricuspid aortic valves (TAVs),^3,4^ with a 50% of probability of requiring aortic valve intervention due to severe AS.^5^

Currently, surgical aortic valve replacement (SAVR) stands as the gold standard for treating AS, albeit being a highly invasive open-heart procedure. To widen the number of patients eligible for treatment, transcatheter aortic valve implantation (TAVI) has emerged as an alternative option, particularly for elderly AS patients with elevated surgical risk. In contrast to surgery, where the native valve is excised, in case of TAVI, the native aortic valve anatomy is more likely to affect valve function and procedural complications.^6^ Theoretically, abnormal cusp fusion, pronounced asymmetry of the valve orifice and annulus, heavily calcified and fibrotic leaflets, and calcified raphe could impair transcatheter aortic valve expansion, eventually leading to paravalvular aortic regurgitation (AR) and poor haemodynamic function.^6,7^ Moreover, partial anchoring and uneven radial forces may lead to annular rupture.^8^

It is evident that in such challenging cases, it is crucial to provide the clinician a comprehensive understanding of specific patient characteristics, to customize the intervention with the aim of reducing the impact of procedural complications. In this context, patient-specific numerical simulations of the TAVI procedure emerge as a powerful tool for gaining predictive insights.

However, several critical aspects, such as the stresses acting on the prosthesis in BAV cases, remain unexplored. Specifically, stress refers to the internal forces exerted within the prosthetic valve structure, which can significantly affect its durability and performance. Excessive stresses may result in mechanical failure, wear, or deformation, ultimately compromising valve functionality. Therefore, stress evaluation is crucial for understanding how the valve will behave under physiological conditions, guiding the selection of appropriate devices and improving long-term outcomes. The absence of such data limits insights into the mechanical response of different devices, hindering informed decision-making regarding optimal valve selection. Moreover, elevated stress levels exerted on the aortic wall have been shown to be associated with post-operative complications, including an increased risk of conduction disturbances.^9^

To overcome this limitation, we employed our validated computational methodology for TAVI procedure^10^ to explore how BAV and TAV valve morphologies affect the selection of prosthetic devices and the potential development of post-operative conduction disturbances, using a novel *in silico* approach.

## Materials and methods

### Study design and population

This is a prospective, single-centre, observational cohort study. Patients undergoing TAVI for severe AS in whom a self-expandable transcatheter heart valve (THV) was implanted at Humanitas Research Hospital (Milan, Italy) were enrolled for the development and validation of a TAVI *in silico* model for predicting post-operative complications. For the purpose of this analysis, only patients diagnosed with Type 1 BAV who underwent TAVI between 2022 and 2023 were included.

### Data acquisition

Clinical, echocardiographic, and procedural data were prospectively collected for all patients, including pre-procedural contrast-enhanced computed tomography (CT) scans and intra-procedural angiographies.

### Modelling process

The anatomies of all subjects, including the left ventricular outflow tract (LVOT), sinuses of Valsalva, coronary arteries, the first tract of the ascending aorta, and calcifications were reconstructed as outlined in our prior publication.^10^ The segmentation was carried out using VMTK commercial software (the Vascular Modeling Toolkit, Orobix, Italy), starting from pre-operative contrast-enhanced CT images. Further details of the patient-specific anatomical model are provided in Supplementary data online, Appendix  *S1*, and shown in Supplementary data online, *Figure S1*. Mechanical properties assigned to the biological tissue components of the model, along with the corresponding literature references, are specified in Supplementary data online, *Table S2*.

ANSA Pre Processor v23.1.1 (BETA CAE System, Switzerland) was used to manually draw the native valve, adhering to the contours of the sinuses of Valsalva.^11^ Specifically, to facilitate the assessment of the influence of various valve morphologies on implantation, both TAV and BAV were reconstructed for each patient (see Supplementary data online, *Figure S2*). In fact, a key element of novelty of this methodology consists in the dual reconstruction of the aortic valve, which was manually delineated in both its bicuspid and virtual tricuspid configurations for comparison, resulting in 10 anatomical models. Details of the BAV model are reported in the Supplementary data online, Appendix  *S2*.

As outlined in our previous study,^10^ a zero-pressure simulation was performed to take in consideration the physiological blood load to which the aorta is subjected. Specifically, during the reconstruction of the patient-specific domain, it is important to account for the fact that the anatomies derived from CT scan segmentations are physiologically loaded by blood pressure, meaning the aortic arterial wall is in a stressed state. CT scans are typically acquired during the end-diastolic phase when blood pressure is approximately 80 mmHg. As demonstrated by Ramella *et al*.^12^ determining an unloaded reference state of the vessel, referred to as the zero-pressure or stress-free configuration, is an essential step to avoid underestimating the aortic stress state. To achieve this, the inverse elastostatic method,^13^ implemented in the ANSYS Mechanical FEA software (Ansys Inc., Canonsburg, PA, USA), was applied to the segmented geometry. These stress-free models served as the initial state for the TAVI simulations.

### TAVI simulations

The self-expandable THV devices, namely, the CoreValve Evolut R (Medtronic, Minneapolis, MN, USA) and the Acurate Neo2 (Boston Scientific, Marlborough, MA, USA), were reconstructed (see Supplementary data online, *Figure S3*) using Solidworks2018 (Dassault Systèmes SolidWorks Corp., Waltham, MA, USA) and subsequently discretized in ANSA. The material properties calibrated in our previous work^10^ were assigned to the nitinol stent.

The workflow previously outlined^10^ was followed for the implementation of TAVI simulations. Briefly, the simulation comprised three steps:

- *Pressurization of the aorta from the zero-pressure configuration to the end-diastolic configuration*. The stress-free configuration was incrementally loaded to 80 mmHg to recreate the blood-loaded geometry.
- *THV crimping inside the insertion catheter.* The prosthetic valve was crimped to a final diameter of 9 mm by the radial displacement of 10 rigid planes and subsequently inserted into the catheter.
- *THV deployment into the aortic root*. The device was deployed inside the aortic root by unsheathing the catheter through a vertical displacement while keeping the prosthesis fixed in place. To accurately replicate the device deployment, two catheters were used for the Acurate Neo2 valve.

Throughout all simulation steps, the nodes at the aortic outlets, including the LVOT, coronary arteries, and the proximal end of the descending aorta, were constrained in all three spatial directions, restricting both displacements and rotations. Penalty contacts between all the components were defined based on the results of a sensitivity analysis.

Simulations were conducted on 28 CPUs of an Intel Xeon64 processor with 250 GB of RAM, using the commercial explicit finite element solver LS-DYNA 971 Release 14.0 (ANSYS, Canonsburg, PA, USA).

To assess the effect of native valve morphology on the implantation procedure, simulations were first conducted on each patient considering the native bicuspid anatomy, and then repeated in a reconstructed tricuspid anatomy, totalling 10 numerical simulations.

### TAVI positioning validation

To validate the accuracy of the simulated positioning of the prosthetic device within the aortic root, a qualitative and quantitative validation of BAV simulations was performed. Specifically, the final configuration of the virtually implanted stents was superimposed on patients’ post-procedural angiographies, allowing a qualitative evaluation of the correct device positioning. To further confirm the accuracy of the simulation with quantitative data, measurements including the distance between the upper crown of the prosthetic device and the aortic wall (Δxs-a), the implantation depth relative to the sinuses of Valsalva (Δz), and the diameter of the lower crown of the device (Ø) were calculated on the angiographic images using ImageJ software. Subsequently, these measurements were compared with the corresponding distances calculated in the simulated implantations.

### Study endpoints

The main objective of the study is to evaluate the effect of different native valves anatomies on prosthetic devices implanted, to identify THV designs that ensure proper TAVI deployment within the aortic root regardless of valve morphology. Our secondary aim is to evaluate the potential increased risk of developing conduction disturbances, annulus rupture, or stent migration depending on patient-specific anatomies. To accomplish these objectives, the analysis focuses on two critical endpoints:

- *Primary endpoint*: The primary endpoint consists of measuring the stresses exerted on the TAVI stent.

Specifically, this measure was repeated across different valvular morphologies for comparison. To determine whether the discrepancy between stress values is solely due to the anatomy of the patient or if it is properly related to the considered device, the implantation of both the Acurate Neo2 and the Evolut R device was simulated in all patients. This assessment is crucial for understanding how these morphologies influence the mechanical integrity and performance of the implanted devices.

- *Secondary endpoint*: As a secondary endpoint, the study aims at measuring the stresses acting on the aortic wall.

To investigate whether the patients’ anatomy affects the distribution and magnitude of stress on the surrounding anatomical structures, a comparison between bicuspid and tricuspid cases was conducted. This analysis is particularly relevant as elevated stresses may lead to conduction disturbances at the His bundle level, with the subsequent need for permanent pacemaker implantation, as well as to annulus rupture. On the other hand, insufficient stress levels could result in THV migration or relevant paravalvular leak.

### Statistical analysis

The Mann–Whitney *U* test was used to assess the differences across the different valve morphologies and simulated THV. This non-parametric test was chosen due to the relatively small sample size and the potential non-normal distribution of variables. Two-sided *P*-values < 0.05 were considered statistically significant. Statistical analyses were performed using Python (version 3.11.6, Python Software Foundation, DE, USA).

## Results

### Patients’ characteristics and modelling

**Figure 1:**
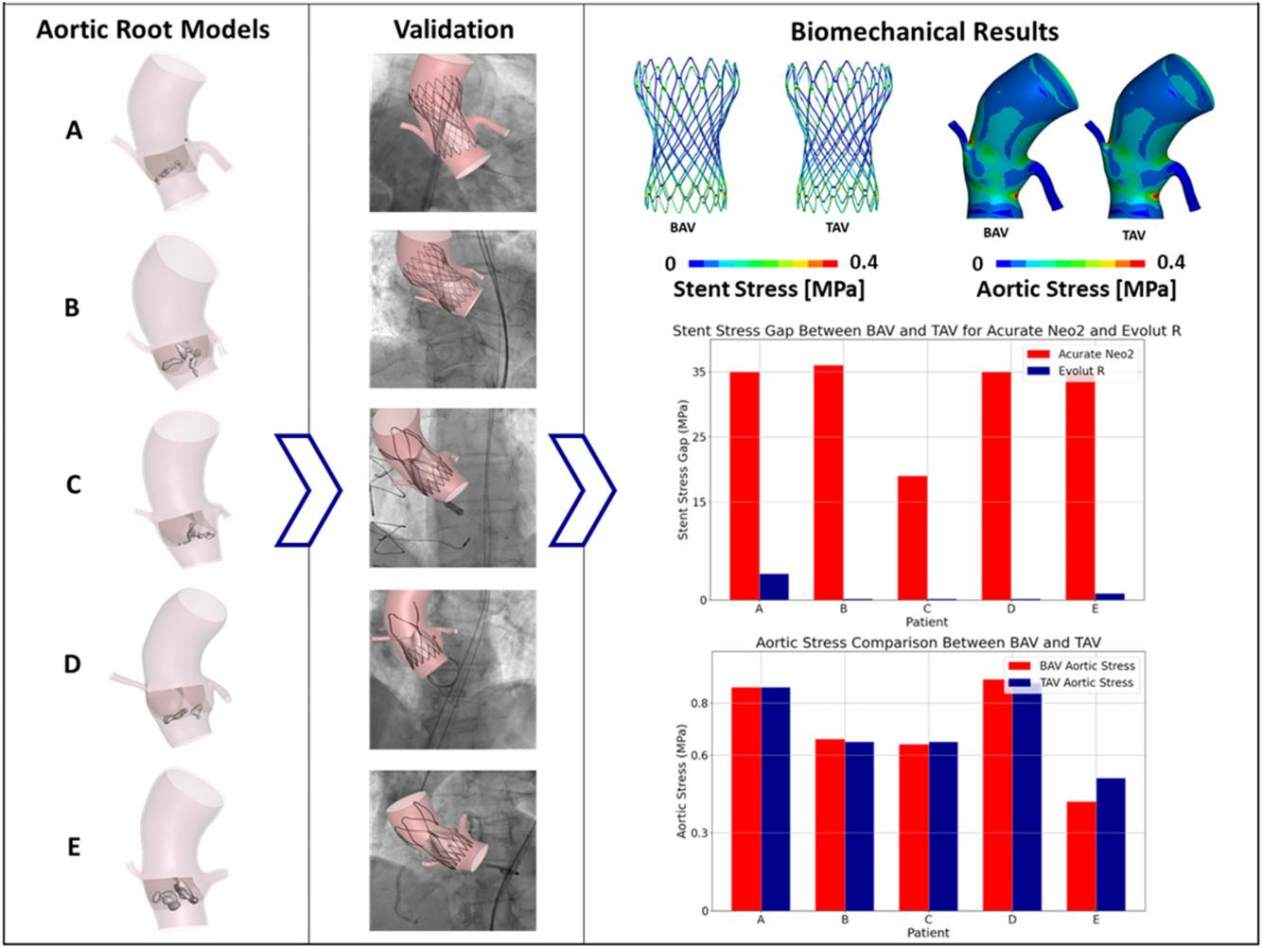
*In silico* study of self-expandable TAVI device selection for BAV patients.

Five patients were enrolled in this study. Baseline features of included patients are reported in *Table 1*. The anatomical models of all enrolled patients can be observed in the *Figure 1*. Numerical details of the reconstructed patients are summarized in Supplementary data online, *Tables S1* and *S2*, respectively.

**Table 1:** Enrolled patients’ echocardiographic and implantation data

	Mean transvalvular gradient (mmHg)	Peak transvalvular gradient (mmHg)	AVA (cm^2^)	TAVI	TAV size
A	38	63	0.9	Evolut R	34
B	74	92	0.6	Evolut R	29
C	26	51	0.8	Acurate Neo2	M
D	46	78	0.9	Acurate Neo2	L
E	63	104	0.8	Acurate Neo2	M

The reported variables are mean and peak pre-procedural transvalvular gradients across the stenotic aortic valve (mmHg), pre-TAVI aortic valve area (AVA, cm^2^), and brand and size of the implanted self-expandable TAV.

M, medium; L, large.

### THV positioning validation

**Figure 2:**
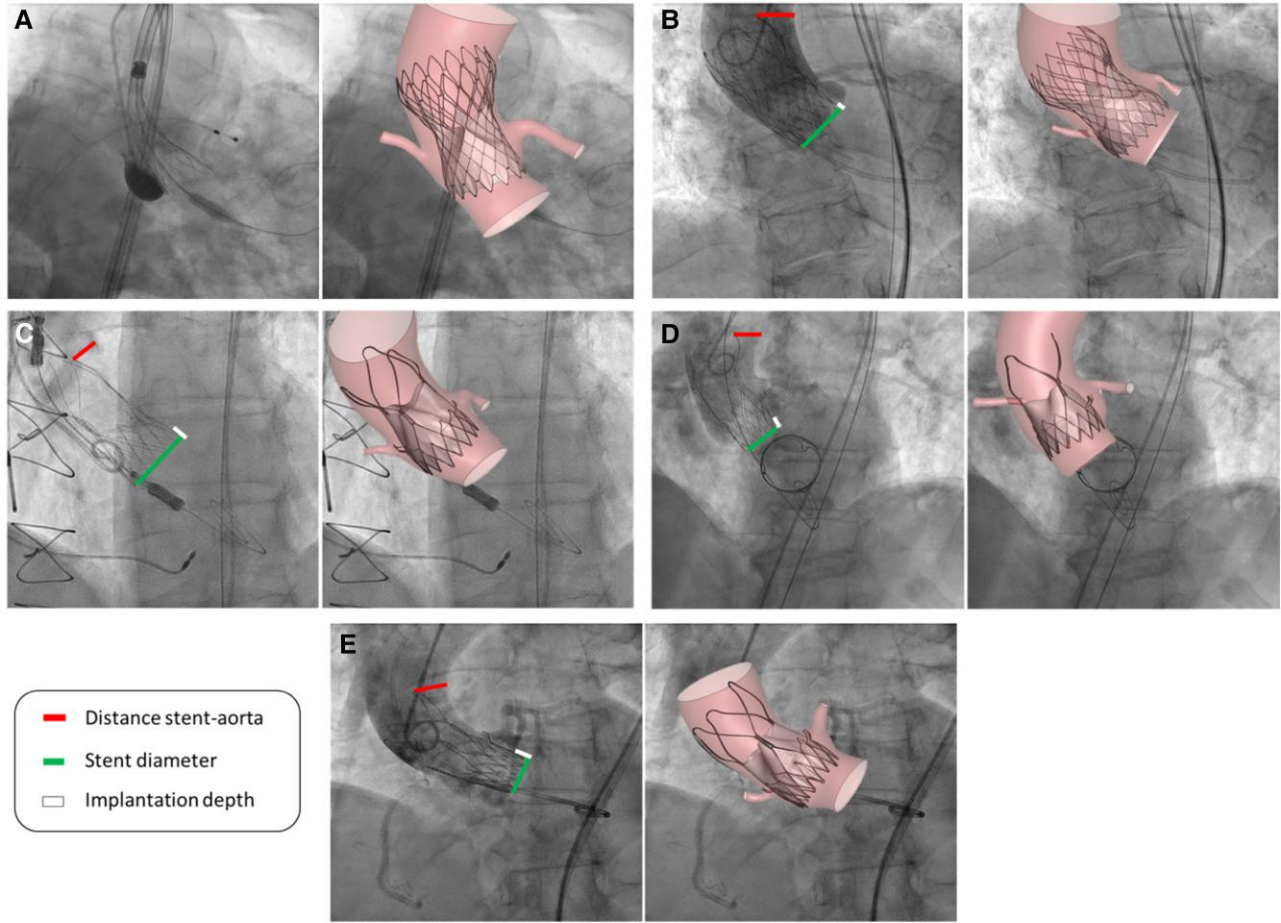
Qualitative validation of TAVI simulations with post-operative angiographies. The distance between the upper crown of the prosthetic device and the aortic wall, the implantation depth relative to the sinuses of Valsalva, and the diameter of the lower crown of the device are reported.

The results of the superimposition of the final configuration of the implanted stents on patients’ post-procedural angiographies are shown in *Figure 2*. The percentage differences between the simulation results and the post-operative CT measurements are detailed in *Table 2*. Measurements for Patient A are not reported because the intra-operative angiography did not provide a clear representation of the fully deployed stent. Notably, a percentage difference lower than 10% was observed in all cases, except for the lower crown diameter of Patient D.

**Table 2:** Percentage difference of the stent–aorta distances, implantation depth, and stent diameter between angiography and simulation

	Δxs-a % difference (%)	Δz % difference (%)	Ø % difference (%)
Patient A			
Patient B	6.01	2.27	6.45
Patient C	1.74	6.99	3.85
Patient D	1.93	5.07	43.25
Patient E	7.34	12.5	7.25

The reported abbreviations represent the distance between the upper crown of the prosthetic device and the aortic wall (Δxs-a), the implantation depth relative to the sinuses of Valsalva (Δz), and the diameter of the lower crown of the device.

### Stresses acting on the THV stent

As reported in *Table 3*, in patients implanted with Acurate Neo2, peak stresses acting on the stent implanted in bicuspid cases are greater than tricuspid ones. On the other hand, this result cannot be confirmed for patients who received the Evolut R device. In fact, it is noticeable that the stress magnitude acting on the prosthetic device assumes comparable values. This result is confirmed when implanting Acurate Neo2 in Patients A and B, where higher stress values were observed in bicuspid cases (*Table 3*), as already identified in Patients C, D, and E. At the same time, the Evolut R TAV was implanted in Patients C, D, and E and, also in these cases, the same maximum stress value was calculated in the bicuspid and tricuspid scenario (*Table 3*). Specifically, in Acurate Neo2 cases, the stress gap between BAV and TAV configurations increased by 21.96 ± 5.35% compared with Evolut R cases (*Figure 1*). Hence, a statistically significant difference in the stress variations between the implanted devices in the BAV and TAV configurations was found (*P* = 0.01). This result highlights the limit of the Acurate Neo2 valve, which solicitation state results much more influenced by the anatomical pattern of the patient, resulting less suitable for bicuspid cases.

**Table 3:** Maximum stress on the stent frame, evaluated reproducing the implantation of both devices (implanted and simulated TAVI) in each patient-specific anatomical model, and on the aortic root in BAV and TAV configurations

		Maximum stent stress (MPa)		Maximum aortic stress (MPa)			Maximum stent stress (MPa)	
	Implanted TAVI	BAV	TAV	BAV	TAV	Simulated TAVI	BAV	TAV
Patient A	Evolut R	287	291	0.86	0.86	Acurate Neo2	281	240
Patient B	Evolut R	284	284	0.66	0.65	Acurate Neo2	184	148
Patient C	Acurate Neo2	149	114	0.64	0.65	Evolut R	135	135
Patient D	Acurate Neo2	123	104	0.89	0.88	Evolut R	348	348
Patient E	Acurate Neo2	228	193	0.42	0.51	Evolut R	165	164

**Figure 3:**
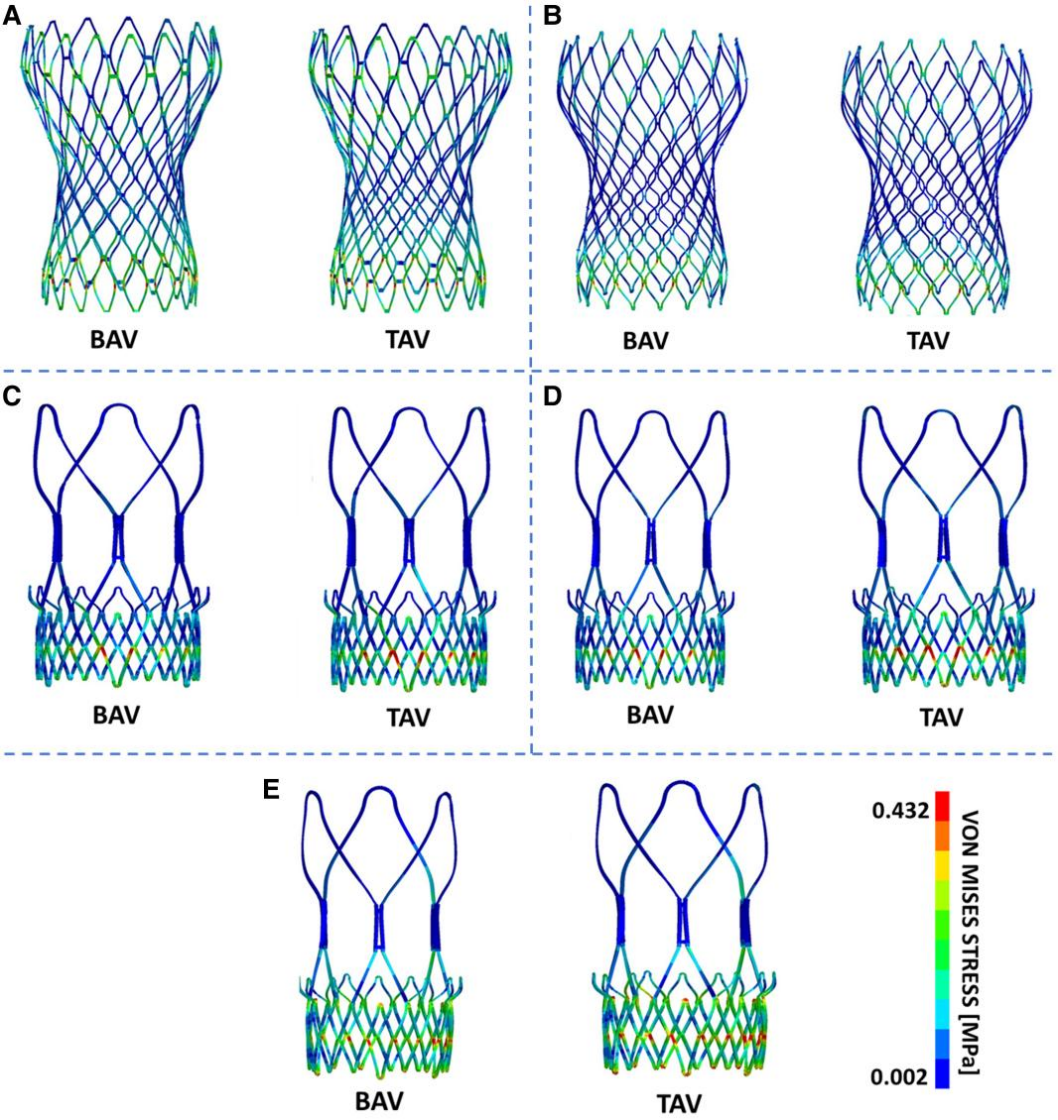
Post-implantation von Mises stress distribution on the stent frames of all enrolled patients (*A–E*). For each patient, both the bicuspid (BAV, left) and tricuspid (TAV, right) configurations are provided for comparison. It can be observed that in all cases, the highest stress values are located at the lower cage of the stent, where the device experiences compression from both the aortic annulus and the native leaflets.

**Figure 4:**
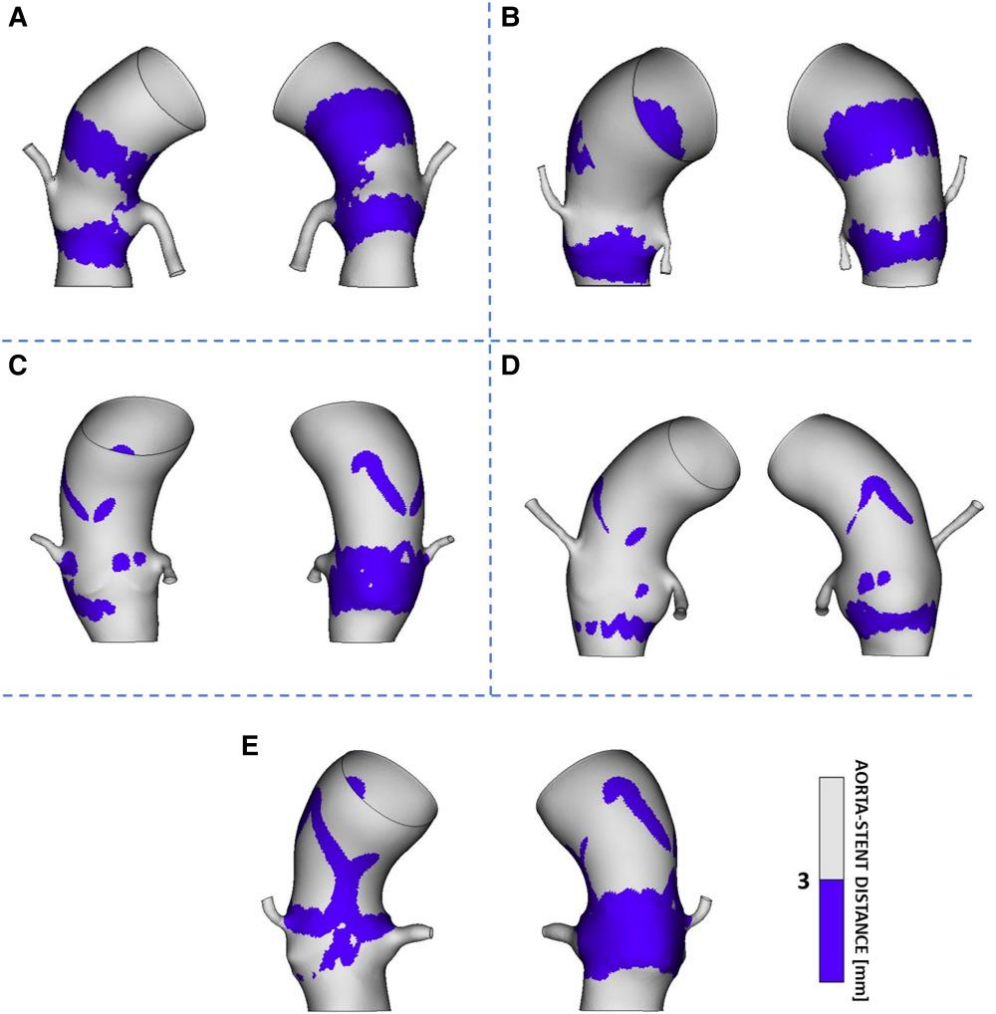
Distance between the stent frame and the aortic wall in enrolled patients (*A–E*). Areas where this value is lower than 3 mm (equal to the sum of the maximum thickness of the stent and the thickness of the aorta) are highlighted on the models. It is noticeable that the lowest distances occur at the level of Valsalva sinuses, where the lower cage of the device is located.

As shown in *Figure 3*, the highest stress values are typically located at the lower cage of the stent, where the device experiences compression from both the aortic annulus and the native leaflets. To support this consideration, the distribution of the distance between the stent and the aorta was computed. As depicted in *Figure 4*, the lowest distances between the stent frame and the aortic wall occur at the level of Valsalva sinuses, precisely at the lower cage of the device.

### Stresses acting on the aortic wall

**Figure 5:**
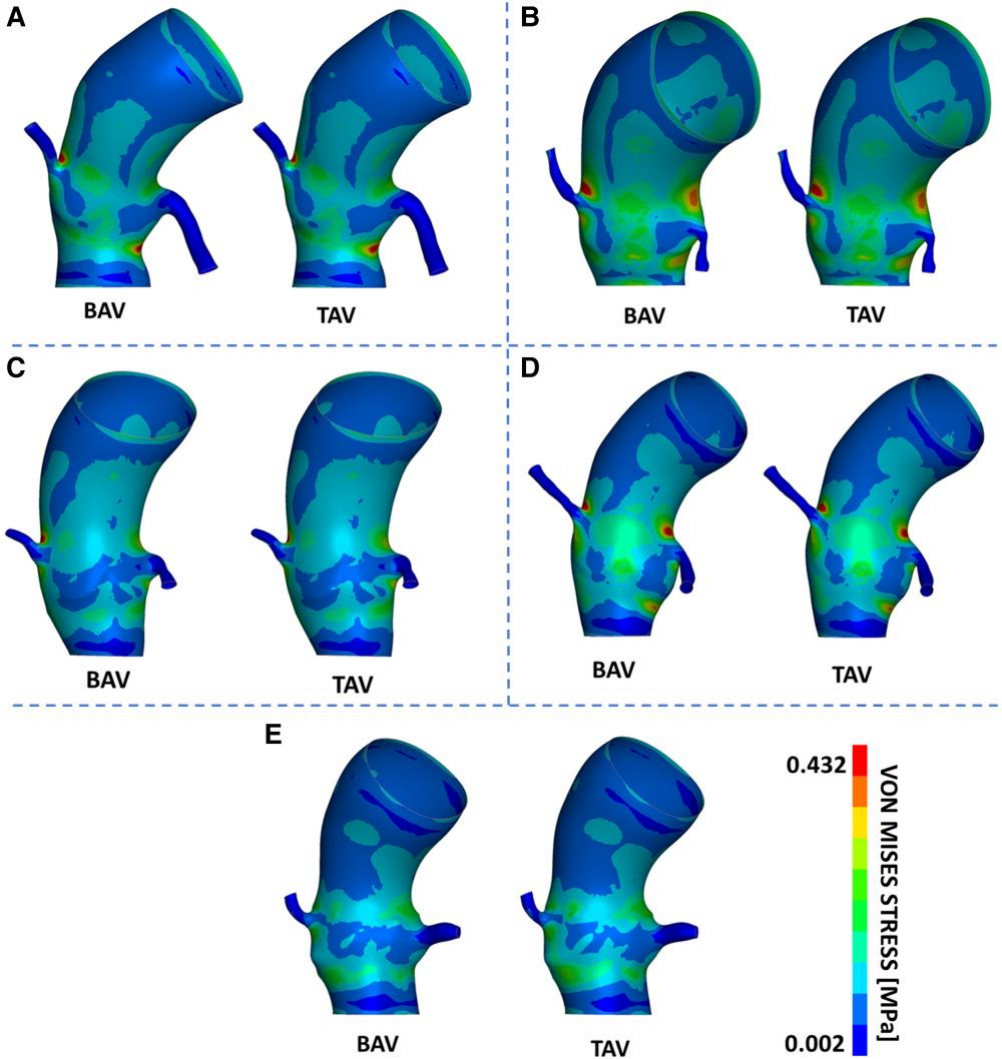
Post-implantation von Mises stress distribution on the aortic wall of all enrolled patients (*A–E*). For each patient, both the bicuspid (BAV, left) and tricuspid (TAV, right) configurations are reported for comparison. Stress distribution and magnitude result comparable, demonstrating that the morphology of the native valve does not impact on implantation-induced aortic stresses.

Across all analysed patients, the distribution of stresses appears consistent between bicuspid and tricuspid native valve cases. Furthermore, as shown in *Table 3*, the comparison of aortic root peak stress values showed no significant differences between BAV and TAV native valve configurations, with a mean stress difference of 5.1 ± 8.17% (*P* = 1.0) (*Figures 1* and *5*).

## Discussion

BAV in patients with AS is often viewed as a relative contraindication for TAVI. This is primarily due to the increased risk of complications associated with this off-label use, leading to their exclusion from major randomized trials, thus limiting the investigation of this pathology. As a result, the adoption of self-expandable THV as a treatment option for BAV patients is still debated. Consequently, several recent clinical studies have attempted to demonstrate the feasibility of implanting self-expandable devices in BAV cases.^14,15^ In this context, computational modelling could hold significant potential, offering complementary insights. However, to the best of the authors’ knowledge, despite the great advances carried out in recent years,^7,8,11,16–24^ some aspects of this approach remain unexplored.

For instance, the stresses acting on the prosthesis in BAV cases have not been investigated yet. This lack hinders insights into the mechanical response of different devices, thereby impeding informed decision-making regarding optimal valve selection for individual patients. Additionally, there is a notable absence of direct comparisons between different self-expandable devices. In a previous work, Finotello *et al*.^22^ compared high deformability (Acurate Neo) and high radial force (Evolut R) devices in four patients, providing valuable analysis on differences in paravalvular orifice area, stress distribution on the aortic root, stent–root interaction area, and stent deformation. Despite their interesting results, the study lacked direct comparison of stress distribution on the stent, and both nitinol frames were modelled with identical material properties, potentially compromising accuracy. Additionally, the reconstruction of both the bicuspid and the virtual tricuspid configuration of the valve was only performed in a previous study from our group,^25^ but it was conducted on a single idealized geometry representing a non-calcified aortic valve. Of note, the lack of reconstruction of the virtual tricuspid configuration prevents from a proper differentiation of valve morphology’s influence from other factors, such as the LVOT eccentricity and calcifications’ pattern, affecting the final stent configuration. Lastly, not all mentioned *in silico* simulations were validated with clinical data, thereby limiting their reliability for translation into clinical practice.

To address these limitations, this paper presents a computational investigation of the influence of different native valve morphologies on TAVI. Specifically, a comparative analysis between implantation in tricuspid and bicuspid Type 1 native valve was performed, assessing the stress magnitude on the final configuration of the implanted stent and the aortic root.

A clinically and experimentally validated numerical framework^10^ was utilized to replicate the TAVI procedure in five patients with Type 1 BAV, guaranteeing a high level of accuracy of simulations. To further validate the accuracy of our simulations, the final configuration of the implanted stents was superimposed on patients’ angiographies, showing a percentage difference lower than 10% in all cases, except for the lower crown diameter of Patient D. This discrepancy is likely due to the pre-dilation performed on that patient, aspect that is not modelled in our simulations. Nevertheless, these results confirm a faithful reproduction of the prosthetic device positioning.

To properly reproduce the anatomical domain, patient-specific anatomical models were reconstructed. Specifically, a key element of novelty consists in the dual reconstruction of the aortic valve, which was manually delineated in both its bicuspid and virtual tricuspid configurations for comparison, resulting in 10 anatomical models. This approach allowed us to distinguish the influence of valve morphology from other factors affecting the stent’s final configuration.

Subsequently, THV implantation of Acurate Neo2 and Evolut R devices was simulated across all cases, allowing a direct comparison between different devices. In particular, for the first time in comparative studies of self-expandable devices in BAV patients, *ad hoc* calibrated material properties of the different stent materials were considered,^10^ ensuring the reproduction of the real mechanical behaviour of the prostheses. This aspect significantly contributes to filling a gap in the current knowledge about the devices’ behaviour in a patient-specific environment, particularly in challenging anatomical scenarios as BAV cases.

Furthermore, the stress distribution and magnitude were evaluated on the aortic root and the final configuration of the implanted stent. This comprehensive analysis provided insights into the impact of native valve morphology on both patient-specific anatomy and THV stent behaviour, thereby introducing novelty to the current state of the art.

Notably, when comparing stent stresses between valvular configurations, Evolut R cases showed comparable stress levels, while Acurate Neo2 implantation in bicuspid cases led to higher stress values, probably due to the reduced and elliptical valve orifice, resulting in greater resistance to the deployment of the stent, thus preventing its complete expansion. Of interest, this trend persisted across all patients, indicating that high radial force devices, such as the Evolut R, are less influenced by native valve morphology, hence resulting more suitable for cases of orifice asymmetry. This finding suggests that Evolut R seems to guarantee consistent TAVI anchoring—due to the Evolut R invariant radial expansion regardless of the native valve morphology—without increasing aortic stress–related complications, such as post-TAVI conduction abnormalities and annulus rupture, and potentially obviating the need of post dilation. In contrast, these results highlight a notable limitation of the Acurate Neo2 valve in cases involving bicuspid anatomy. In fact, the Acurate Neo2’s performance seems more heavily influenced by the patient’s specific anatomical characteristics, resulting in increased sensitivity to the native valve morphology. This susceptibility can lead to uneven stress distribution, especially in BAV cases, where the valve structure is inherently asymmetrical. Such uneven stress distribution may result in suboptimal expansion and incomplete sealing, thereby increasing the risk of paravalvular leakage. Moreover, higher stress levels in specific areas can contribute to mechanical strain on the valve material, adversely affecting its durability.

Furthermore, the analysis of stresses on the aortic root revealed no differences between different native valve configurations or prosthetic devices, demonstrating that the morphology of the native valve does not impact on implantation-induced aortic stresses. This result implies that the implantation of self-expandable THV in BAV patients does not seem to translate into a heightened risk of post-TAVI conduction abnormalities or annulus rupture, regardless of the selected device.

While this study provides valuable insights, a few limitations must be stated. First, the small sample size of five patients, resulting in 10 anatomical models, as well as the single-centre nature of the study, limit the generalizability of the findings. This study was intended as a proof-of-concept, demonstrating the feasibility and potential of patient-specific *in silico* modelling for meticulously evaluating device performance and ensuring patient safety in complex cases, such as those involving BAVs. To address this limitation, future research will include larger, multicentre cohorts to enhance statistical power and applicability. Additionally, the study focuses on immediate mechanical outcomes rather than long-term clinical effects, such as leaflet durability or paravalvular leak progression. While these factors were not the primary focus of this paper, it is clear that evaluating long-term clinical implications is crucial for a comprehensive understanding of the lasting impact of TAVI devices. This limitation will be addressed in future analyses, which will incorporate extended clinical data to establish meaningful correlations with simulation results. The modelling approach also simplifies certain procedural aspects, such as excluding balloon pre- and post-dilation, and relies on end-diastolic anatomical configuration rather than fully dynamic simulations. Future studies will also integrate fluid–structure interaction simulations to capture the dynamic behaviour of the valve and blood flow, allowing for more accurate procedural representation and better assessment of long-term device performance. Furthermore, this work specifically examines only Type 1 BAV morphology due to its higher data availability, as it represents the most common BAV subtype. Future research should expand to other BAV subtypes to provide a more comprehensive understanding of TAVI device performance across different anatomical configurations. Lastly, our findings are specific to the Evolut R and Acurate Neo2 devices, which have distinct mechanical properties and expansion characteristics. While these results provide valuable insights, generalizing them to other TAVI devices, especially newer or alternative self-expandable devices, should be done cautiously. Future studies will be necessary to evaluate the performance of additional devices with different stent designs and mechanical properties. This could involve repeating material property calibration with experimental tests and reapplying the analysis to other TAVI devices, which would help extend the applicability of our findings.

Despite these limitations, this research establishes a robust foundation for further investigations and highlights the potential of *in silico* models for TAVI device evaluation in complex clinical scenarios. Through the achieved results, this study underlines the importance of numerical simulations in BAV cases, particularly in the selection of the appropriate bioprosthetic valve. By quantitatively evaluating the influence of valve morphology on patient and device stress level, these simulations offer valuable insights for clinical decision-making. Incorporating this validated methodology into the pre-operative planning could support clinicians in the case-by-case selection of the most suitable prosthetic device, thereby significantly reducing the risk of potential complications in the daily clinical practice.

This study demonstrates that high radial force devices, such as the Evolut R, provide consistent expansion in BAV cases, rendering them more suitable for such patients. Notably, stresses on the aortic root result comparable across different valvular morphologies, suggesting that the Evolut R ensures stable TAVI anchoring without significantly increasing the risk of aortic stress–related adverse events, such as conduction abnormalities and annulus rupture. Incorporating this validated methodology into pre-operative planning could support clinicians in selecting the most suitable device with a patient-specific approach.

## Supplementary data

Supplementary data are available at European Heart Journal – Imaging Methods and Practice online.

## Consent

The research protocol received approval from the Institutional Ethics Committee, ensuring compliance with ethical guidelines and standards. All participating patients provided informed, written consent, confirming their voluntary participation. Prior to enrolment, each patient received a comprehensive explanation of the study’s purpose, procedures, and potential benefits. All identifying information has been anonymized to maintain patient confidentiality in accordance with institutional requirements and ethical guidelines.

## Supplementary Material

qyaf018_Supplementary_Data

## Data Availability

The data underlying this article will be shared on reasonable request to the corresponding author.

## Lead author biography

Benedetta Grossi is a biomedical engineer and PhD student in Data Science in Medicine at Humanitas University. She holds a degree in Biomedical Engineering from Politecnico di Milano, where she is also a visiting PhD student. Her research focuses on *in silico* modelling for cardiovascular applications, with a particular emphasis on transcatheter aortic valve implantation. After gaining professional experience in the field of cardiovascular medical devices and building a strong background in computational biomechanics and data science, Benedetta collaborates with interdisciplinary teams to translate theoretical models into actionable insights for clinicians, with the ultimate goal of optimizing outcomes for patients with complex cardiovascular conditions.
