# Supplementary material for: The impact of bicuspid valve morphology on the selection of transcatheter aortic valve implantation devices: an in silico study
Source: Eur Heart J Imaging Methods Pract. 2025 Feb 5;3(1):qyaf018. doi: 10.1093/ehjimp/qyaf018 (PMC11879518; doi:10.1093/ehjimp/qyaf018)
Supplement: qyaf018_Supplementary_Data [file qyaf018_supplementary_data.docx]

**Supplementary Data**

**Supplementary Appendix 1. Details of the patient-specific anatomical model**

Patient-specific anatomical models – including left ventricular outflow tract (LVOT), sinuses of Valsalva, the first tract of the ascending aorta, coronary arteries inflow and calcifications – were reconstructed from pre-operative contrast-enhanced CT scans (**Supplementary Figure 1a and 1b**), using VMTK software (The Vascular Modeling Toolkit, Orobix, Italy). After the reconstruction, the anatomical models were pre-processed using both VTMK and Meshmixer (v3.5, Autodesk Inc, US). In particular, VMTK was employed to create inlet and outlet surfaces of the domain of interest, while both software packages were used to smooth the geometry, aiming to remove noise and motion artefacts. After that, excessively short edges or sharp angles were removed in ANSA Pre Processor v23.1.1 (BETA CAE System, Switzerland). Since it could not be detected from CT scans, ANSA was also used to manually draw the leaflets of the patients’ native valve (**Supplementary Figure 1c**). The leaflets were generated following the profile of the sinuses of Valsalva, attempting to reproduce their real shape. A section thickness equal to 2.1 mm and 0.1 mm was assigned to the aortic root and the native leaflets, respectively.

**Supplementary Figure**
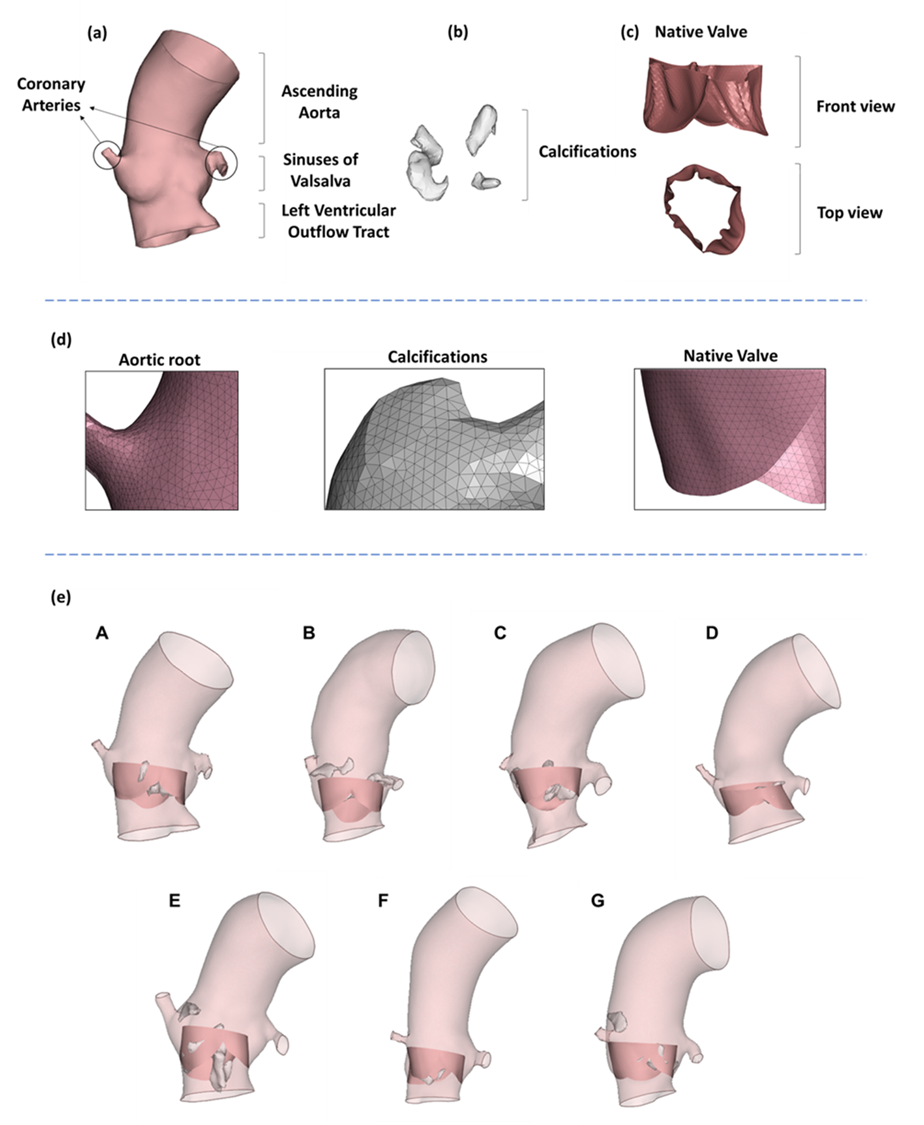
**1**

**Supplementary Figure 1.** Exemplificative patient-specific anatomical model. (a) Aortic root reconstruction, including left ventricular outflow tract, sinuses of Valsalva, first tract of the ascending aorta and coronary arteries; (b) calcifications; (c) front and top view of the reconstructed native aortic valve.

**Supplementary Figure 2**


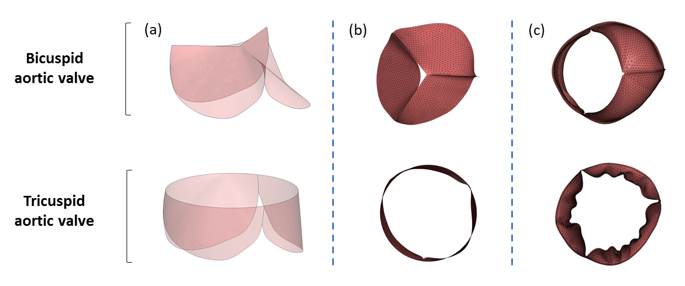


**Supplementary Figure 2.** Front (a) and top (b) views of the bicuspid (upper panel) and tricuspid (lower panel) aortic valve initial reconstructions. The tricuspid valve is represented in its open configuration, while the bicuspid valve is depicted in its closed state. Panel (c) shows the configuration utilized as the starting point for TAVI simulations, depicted in its top view.

**Supplementary Appendix 2. Details of the BAV model**

To ease the CAD drawing, the tricuspid valve was modelled in its open configuration, while the bicuspid valve was depicted in its closed state (**Supplementary Figures 2a and 2b**). To faithfully replicate the anatomy of a patient-specific type I BAV, the following steps were implemented:

1. A circumference passing through three points at the level of the coronary ostia was drawn, accompanied by three curved lines delineating the contours of the aortic sinuses, and three vertical lines connecting the vertices of the curves to the circumference;
2. Curved and vertical lines outlining the leaflets profile were projected onto the aortic mesh;
3. Three straight lines from the terminal node of the projected vertical lines to the centre of the circumference were drawn and discretized with beam elements;
4. Within the area delimited by the projections and the aforementioned straight lines, three surfaces were generated to replicate the closed configuration of the three leaflets;
5. The nodes along the free margins of the two fused leaflets were merged.

Prior to the implantation procedure, a preliminary simulation was conducted to open the orifice of the bicuspid aortic valves, which were initially depicted in their closed configuration. This step aimed to create adequate space for the subsequent catheters’ insertion. Specifically, a pressure ramp was applied to the internal surface of the valve elements, and the final frame of the simulation was extracted. To replicate the same orifice opening in the tricuspid configuration, a pressure ramp was also applied to the outer elements of the tricuspid valve to partially close it. The initial geometries of the valves’ configurations used for the TAVI simulation are illustrated in **Supplementary Figure 2c**.

**
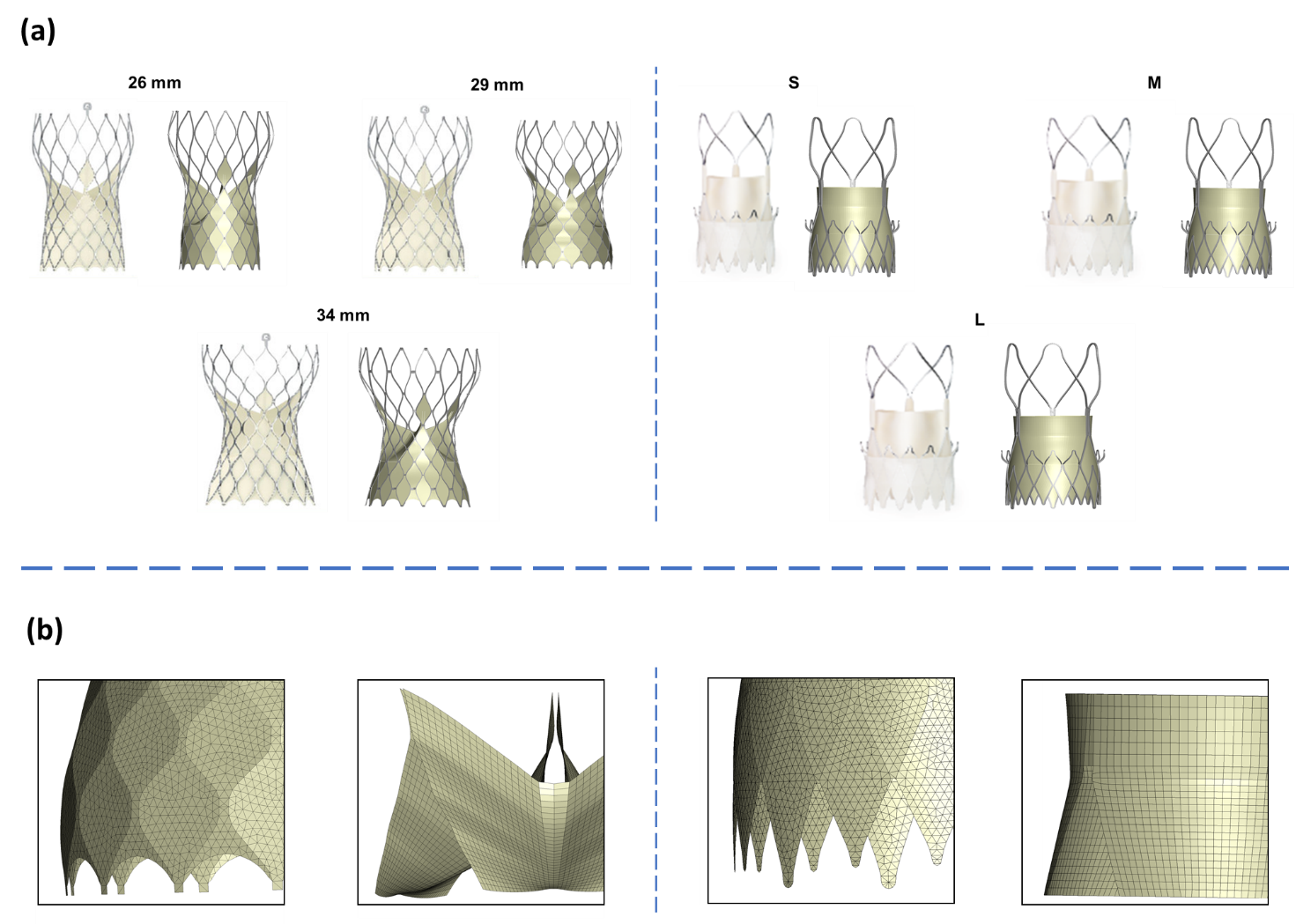
Supplementary Figure 3**

**Supplementary Figure 3.** (a) Representation of the library of reconstructed self-expandable bioprostheses. Specifically, on the left the Evolut R TAV is reported in three sizes (26 mm, 29 mm and 34 mm), while in the right panel the Acurate Neo2 valve is shown in its three versions (S – 23 mm, M – 25 mm and L – 27 mm); (b) Mesh details.

**Supplementary Table 1. Mesh element details for (A-E) patient-specific anatomical models**

**(Δx: element size).**

|  | **Number of Elements** | | | |
| --- | --- | --- | --- | --- |
|  | **Aorta**  (Δx = 0.8 mm)  triangular shell elements | **Tricuspid aortic valve**  (Δx = 0.8 mm)  triangular shell elements | **Bicuspid aortic valve**  (Δx = 0.8 mm)  triangular shell elements | **Calcifications**  (Δx = 0.5 mm)  tetrahedral solid elements |
| **A** | 34687 | 3658 | 3946 | 5395 |
| **B** | 30294 | 3624 | 3851 | 13635 |
| **C** | 32026 | 3880 | 4212 | 9559 |
| **D** | 42433 | 5755 | 6439 | 13284 |
| **E** | 26421 | 3057 | 3365 | 28826 |

**Supplementary Table 2. Mechanical properties assigned to the model components with the corresponding literature references.**

| **Component** | **Material model** | **Material parameters** | **Reference** |
| --- | --- | --- | --- |
| Aortic root | Yeoh - 3^rd^ order | C10 = 0.041 MPa  C20 = 0.118 MPa  C30 = 0.455 MPa | 2012, Azadani et al. |
| Native leaflets | Mooney-Rivlin 3 | C10 = 0.033 MPa  C01= 0.0023 MPa  C11 = 0.5858 Mpa | 2020, Pasta et al. |
| Calcifications | Linear elastic | E=12.6 MPa  ν=0.45 | 2004, Holzapfel et al. |
